# Impact of process stress on protein stability in highly-loaded solid protein/PEG formulations from small-scale melt extrusion

**DOI:** 10.1016/j.ijpx.2022.100154

**Published:** 2022-12-30

**Authors:** Katharina Dauer, Christian Werner, Dirk Lindenblatt, Karl Gerhard Wagner

**Affiliations:** a University of Bonn, Department of Pharmaceutics, Institute of Pharmacy, Bonn, Germany; b University of Cologne, Department of Chemistry, Institute of Biochemistry, Cologne, Germany

**Keywords:** Hot-melt extrusion, Protein stability, Solid-state characterization, Small-scale, BSA, bovine serum albumin, BSE, backscattered electron, CD, circular dichroism, DSC, Differential Scanning Calorimetry, EDX, energy-dispersive X-ray detector, EVA, Ethylene-vinyl acetate, FTIR, Fourier transformation infrared spectroscopy, HME, hot-melt extrusion, HMWS, high molecular weight species, PEG, polyethylene glycol, PEO, polyethylene oxide, PLGA, Poly Lactic-co-Glycolic Acid, SEM, scanning electron microscopy, ss, solid-state, TSE, twin-screw extrusion

## Abstract

As protein-based therapeutics often exhibit a limited stability in liquid formulations, there is a growing interest in the development of solid protein formulations due to improved protein stability in the solid state. We used small-scale (<3 g) ram and twin-screw extrusion for the solid stabilization of proteins (Lysozyme, BSA, and human insulin) in PEG-matrices. Protein stability after extrusion was systematically investigated using ss-DSC, ss-FTIR, CD spectroscopy, SEM-EDX, SEC, RP-HPLC, and in case of Lysozyme an activity assay. The applied analytical methods offered an accurate assessment of protein stability in extrudates, enabling the comparison of different melt extrusion formulations and process parameters (e.g., shear stress levels, screw configurations, residence times). Lysozyme was implemented as a model protein and was completely recovered in its active form after extrusion. Differences seen between Lysozyme- and BSA- or human insulin-loaded extrudates indicated that melt extrusion could have an impact on the conformational stability. In particular, BSA and human insulin were more susceptible to heat exposure and shear stress compared to Lysozyme, where shear stress was the dominant parameter. Consequently, ram extrusion led to less conformational changes compared to TSE. Ram extrusion showed good protein particle distribution resulting in the preferred method to prepare highly-loaded solid protein formulations.

## Highlights

• Useful approaches for investigation of protein stability in melt extrudates.
• Successful distinction of melt extrusion processes and extrusion parameters.
• Increased susceptibility of BSA and insulin to shear stress compared to Lysozyme.
• Ram extrusion led to less conformational changes compared to TSE.
• Interactions between hydrophilic PEG chains and insulin without stabilizing effect.

## Graphical Abstract

**Figure:**
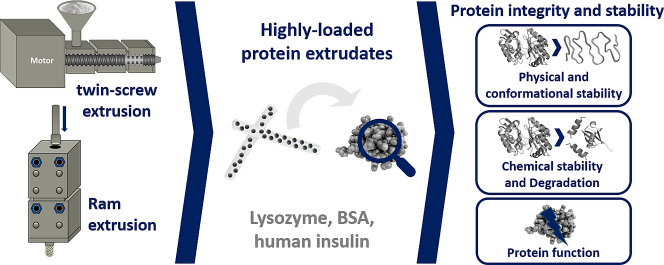

## 1 Introduction

Since biopharmaceutics play a steadily increasing role in the medical treatment of especially severe diseases, the sector of developing protein-based therapeutics is similarly growing. Biopharmaceutics are more complex and have to be administered mostly parenteral as liquid formulations compared to other drug products based on small-molecule drugs (Gervasi et al., 2018). However, the demand of liquid formulations for application is in contradiction to an often limited chemical and physical stability in liquid state (e.g., formation of aggregates) which is considered as major challenge for the successful development and lifecycle of a biopharmaceutical drug product (e.g., manufacturing, formulation development, processing, storage, and shipping) (Mahler et al., 2009; Roberts, 2014). The formation of protein aggregates in liquid formulations can lead to an increased immunogenicity, viscosity, and injection force. Further aggregation leads to countable sub-visible or visible particles, which are both not accepted by the authorities (Mahler et al., 2009; Ratanji et al., 2014; Jiskoot et al., 2012; Pham and Meng, 2020; Fischer et al., 2015). Most of the analytical methods applied during protein formulation development studies focus on the investigation and characterization of proteins in solution or lyophilized proteins after their reconstitution. Analytical characterization methods for liquid protein formulations include: (i) RP-HPLC for the analysis of degradation products and chemical stability, (ii) size exclusion chromatography (SEC) for monitoring the loss of protein monomers as an indication of aggregate formation, (iii) circular dichroism (CD) and nuclear magnetic resonance (NMR) to probe conformational stability and (iv) light scattering methods for estimating the colloidal stability of protein formulations and monitoring the appearance of sub-visible particles (Capelle et al., 2007; Chaudhuri et al., 2014; Dauer et al., 2021a; Dauer et al., 2021b; Bhirde et al., 2020; Ma et al., 2020).

In contrast, solid formulations of proteins offer the potential of increased stability (Al-Azzam et al., 2002; Rezvankhah et al., 2020). Proteins are commonly freeze-dried to overcome the instability issues that may occur in liquid protein formulations. Thus, there is a growing interest in the development of other solid protein formulations (e.g., protein-loaded extrudates or implants) as well as encapsulation procedures that employ dehydrated protein powders due to improved protein stability in the solid state. During lyophilization, proteins are molecularly dispersed primarily in an amorphous excipient matrix resulting in an increased stability (Wang et al., 2010). Nowadays, there is an increasing awareness to use melt extrusion processing for the preparation of solid protein-loaded implants or encapsulation of protein particles in extrudates with focus on solid-state protein stabilization. Melt extrusion is used in pharmaceutical industry as continuous and robust manufacturing tool for the preparation of solid dosage forms. Melt extrusion (i) can result in the embedment of drugs in polymeric matrices, (ii) is a solvent-free (e.g., organic solvents) technique, and (iii) does not require additional excipients such as surfactants or cryoprotectants (Gajda et al., 2018; Madan and Madan, 2012). Melt extrusion processing is also applied for the embedding of drug particles or drug crystals at high drug loadings (up to 80%) as well as for the preparation of amorphous solid dispersions, where the drug is dispersed on a molecular level in a polymeric carrier (Prodduturi et al., 2005; Simons and Wagner, 2019; Özgüney et al., 2009). Additionally, small-scale melt extrusion is accompanied with (i) processing small batch sizes (e.g., less than 2 g of a protein formulation), (ii) high yield, and (iii) short process times (e.g., shorter than 3 min for 2 g of a powdered protein formulation). The major drawbacks of commonly used techniques such as spray- or freeze-drying to formulate or encapsulate proteins in solid-state are mostly (i) the use of low-concentrated protein solutions resulting in a limited powder yield, (ii) relatively long processing times, (iii) the need of disaccharides or surfactants in the aqueous solution to prevent protein aggregation or inactivation, and (iv) the limited process scalability (Butreddy et al., 2020; Assegehegn et al., 2019; Assegehegn et al., 2020; Kasper et al., 2013). Furthermore, the use of polymers as stabilizing excipients are not or difficult to be processed via spray-drying due to the rheological behavior of the spray solution and is strongly depended on the polymer used. Phase separation of polymers or the crystallization of other excipients and formulation components during spray-drying may lead to denaturation or the formation of protein aggregation (Assegehegn et al., 2020; Heller et al., 1997; Al-Hussein and Gieseler, 2012). However, the spray solution rheology depends not only on the polymer but also on the solvent system and their interaction, and thus determines the sprayability (Porfirio et al., 2020). On the other hand, during freeze-drying, the formulations which contain amorphous saccharides (e.g., sucrose), are prone to collapse upon the primary drying step and the collapse is unfavorable and often negatively affects the storage stability of proteins (Haeuser et al., 2019). In contrast, in melt extrusion processing, the polymers can be easily used to embed proteins in a polymeric matrix without dissolution of the protein particles in the polymer for solid-state stabilization, however, dry protein powders are required for melt extrusion processing. In this case, the protein particles act as fillers resulting in a reduced amount of energy required for the viscous dissipation during melt extrusion processing (Lyons et al., 2006). Polymers can ensure the thermodynamically stable folded structure of a protein during the melt extrusion process due to the immobilization of protein molecules in the polymeric matrix, which is critical for a successful protein formulation development (Ghalanbor et al., 2010; Lee et al., 2006; Zheng and Pokorski, 2021). Furthermore, by melt extrusions processing a high fraction of protein can be embed into a polymeric matrix resulting in high-drug loads (i.e., >40% protein) compared to other formulation techniques. Another benefit of hot-melt extruded solid protein formulations compared to lyophilized proteins is the reduced static electricity. Often, the static electricity of lyophilized protein powder is so severe that it is impossible to handle it safely (e.g., weighed-in inaccurately, problems during sample preparation for analytical investigation) (Takada et al., 2003).

However, during melt extrusion processing protein exposure to elevated temperatures and shear stress can potentially cause unfolding and degradation, leading to irreversible aggregation and/or a loss of functionality and is also a factor that should not be underestimated. Especially, information on an intensive assessment of protein stability in solid formulations prepared by melt extrusion processes and the impact of process stress is not as widely reported. In this study, we focused on the characterization of highly-loaded protein extrudates (i.e., 40% protein content) prepared by ram extrusion and twin-screw extrusion (TSE) with regard to the main challenges, i.e., protein instability due to heat exposure (Lai and Topp, 1999) and shear stress during extrusion (Manning et al., 2010). The aim was the implementation of a sensitive characterization pathway of protein stability in highly-loaded solid protein/PEG formulations. Lysozyme, bovine serum albumin (BSA), and human insulin were chosen as (model) proteins and peptide, respectively and were embedded in polyethylene glycol (PEG) 20,000-matrices. PEG is a hydrophilic polymer and next to an increasing half-life of the protein also used as potential protein stabilizer by addition of PEG-chains to proteins (PEG-ylation) or co-processing with PEG (Al-Azzam et al., 2002; Cantin et al., 2021; Chen et al., 2021a). Furthermore, several studies suggest that long PEG-chains (e.g., PEG 20,000) can stabilize proteins by interacting with the protein surface or by the formation of the excluded volume effect to decelerate the unfolding rate of the protein (Chao et al., 2014; Pandey et al., 2013). Furthermore, higher molecular weight PEG (also known as polyethylene oxide (PEO) depending on its molecular weight) offers an potential as matrix polymer for controlled release dosage forms and is commercially available in a broad variety of PEO grades and different molecular weights (e.g., 100,000, 300,000, 1,000,000) (Cantin et al., 2021). We selected PEG 20,000 as model polymer due to its (i) low melting temperature (∼60 °C, depending on the chain-length), (ii) hydrophilicity and (iii) potential as protein stabilizer. Due to the hydrophilic character of PEG 20,000, we favored a solely embedment and dispersion of the protein particles in the polymeric matrix without dissolution of the protein particles in the polymer. Furthermore, the hydrophilicity of PEG enormously simplified the sample preparation and thus the analytical investigations. More specific, the development and implementation of a complex protein extraction protocol was not necessary. Lysozyme is a commonly used model protein in pharmaceutical and biological research (Ghalanbor et al., 2010; Mohammad et al., 2015; Elkordy et al., 2002; Sheng et al., 2016) and was used for the implementation of analytical procedures in the sense of a negative control. The protein stability and integrity of Lysozyme, BSA and human insulin after melt extrusion was systematically investigated using (i) RP-HPLC (chemical stability and protein concentration), (ii) SEC (protein fragment and aggregation analysis), (iii) SEM-EDX (protein particle distribution), (iv) DSC (protein's unfolding temperature), (v) FTIR and CD spectroscopy (conformational stability), and (vi) activity assay. We provide characterization methods to assess process-related protein stability in highly-loaded protein extrudates as well as to distinguish between the impact of different small-scale melt extrusion processes (i.e., ram and TSE) and extrusion parameters (e.g., shear stress levels, screw configurations, residence times) on protein stability. We considered melt extrusion for the production of protein-loaded extrudates in order to further enhance their stability in solid-state formulations compared to the starting material, for improved storage and logistic conditions. Another perspective would be the production of protein-loaded implants with sustained release profile. The proposed characterization pathway for proteins processed via small-scale melt extrusion proved to be a powerful tool to assess the formulation stability in PEG-matrices and potentially other excipients like PEO, lipids, poloxamers, Poly Lactic-*co*-Glycolic Acid (PLGA), and Ethylene-vinyl acetate (EVA) (Ghalanbor et al., 2010; Zarrintaj et al., 2020; Hamoudi-Ben Yelles et al., 2017; Ghalanbor et al., 2012; Cossé et al., 2017; Even et al., 2017; Stanković et al., 2015; Stanković et al., 2013; Buske et al., 2012). The two latter would subsequently lead to the development of long releasing implants and will be discussed in future works (Cossé et al., 2017; Vollrath et al., 2017). Furthermore, the implemented methods should be expand to or combined with other formulation and stabilization techniques (e.g., vacuum compression molding, a combination of spray-drying and melt extrusion) in order to study process-related effects on protein stability.

## 2 Materials and methods

### 2.1 Materials

Lysozyme from chicken egg-white, lyophilized powder (Cat. No. L6876, d_50_ = 137 μm) was obtained from AppliChem (AppliChem GmbH, Darmstadt, Germany). Bovine serum albumin (BSA) (d_50_ = 505 μm), sodium chloride, di‑sodium hydrogen phosphate dihydrate, sodium dihydrogen phosphate dihydrate, acetonitrile and trifluoroacetic acid (HPLC gradient grade) were purchased from Merck (Merck KGaA, Darmstadt, Germany). Human insulin (d_50_ = 141 μm) was kindly donated by Sanofi-Aventis Deutschland GmbH (Frankfurt, Germany). Polyethylene glycol 20,000 was obtained from Carl Roth (Carl Roth GmbH & Co. KG, Karlsruhe, Germany).

### 2.2 Preparation of protein-loaded PEG-extrudates by ram and twin-screw extrusion

**Fig. 1:**
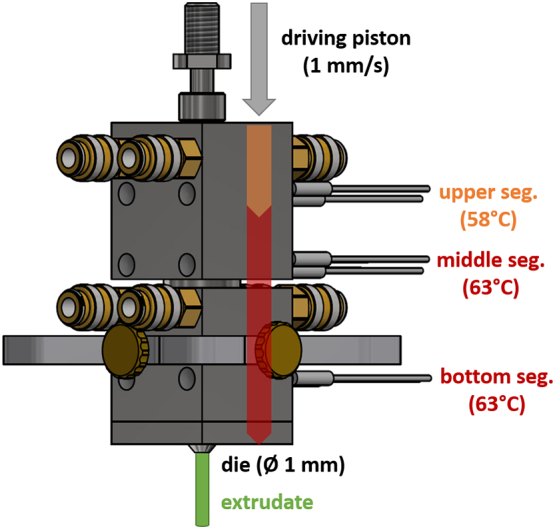
Self-built ram extruder. Barrel (10 cm length, inner cylindrical hole Ø = 10 mm) consisted of three heating zones and a cylindrical die (Ø = 1 mm). Upper temperature segment (filling-zone): 58 °C, middle segments were set to 63 °C. The mixture was heated for three minutes and the molten blends were subsequently extruded through the 1 mm-die by a driving piston with a speed of 1 mm/s.

**Fig. 2:**
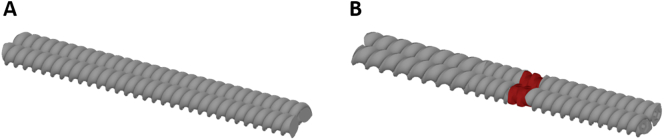
Screw configurations of the 5 mm TSE with: A conveying elements (5 mm pitch), B a single kneading element (7.5, 5 mm pitch, 90° kneading element (red), 5 mm pitch); Segment 1 (ambient temperature), segment 2 (63 °C), and segment 3 (60 °C).

PEG 20,000 flakes were milled utilizing a high-shear mixer (Krups Mixette type 210, Krups, Frankfurt am Main, Germany) and passed through a 650 μm sieve to remove larger particles. For the preparation of protein-loaded extrudates, a physical mixture (3 g) composed of 60% PEG 20,000 and 40% protein powder (Lysozyme, BSA, or human insulin) were blended for 10 min at 50 rpm using a turbula mixer (Willy A. Bachofen AG, Muttenz, Switzerland). The physical mixtures of PEG 20,000 and protein powder (Table 1) were either ram extruded (lower shear stress) or hot melt extruded by using TSE (higher shear stress). We used a self-built ram extruder for the preparation of protein-loaded extrudates with lower shear stress (Fig. 1). A barrel of 10 cm length and an inner cylindrical hole of 10 mm diameter consisted of three heating zones and was equipped with a cylindrical die (diameter 1 mm). Extrudates were prepared by feeding 3 g of the physical mixture into the 10-mm hole of the ram extruder. The temperature of the upper segment (filling-zone) was set to 58 °C, and the following two segments were set to 63 °C. The mixture was heated for three minutes and the molten blends were subsequently extruded through the 1 mm-die by a driving piston with a speed of 1 mm/s. TSE was performed using a 5 mm co-rotating twin-screw extruder ZE 5 (Three-Tec GmbH, Seon, Switzerland) with a functional length of 15:1 L/D and two heating zones, equipped with a 1 mm die and either a conveying screw configuration, or a screw configuration with a single kneading element, which are presented in Fig. 2. The physical mixtures (3 g) were fed into the extruder by using a powder belt conveyor (GUF-P Mini AD / 475 / 75, mk Technology Group, Troisdorf, Germany) at constantly kept feeding rates of 0.4 g/min or 0.8 g/min ± 5% for Lysozyme, human insulin, or BSA, respectively (Table 1).

**Table 1:** Overview of the compositions and prepared protein-loaded extrudates by ram or twin-screw extrusion with applied process parameters; the physical mixtures were prepared in a turbula mixer at a frequency of 50 rpm for 10 min.;for ram extrusion the physical mixture was heated for 3 min. at 63 °C and extruded with a piston speed of 1 mm/s through a 1 mm-die; for TSE the physical mixture was constantly fed (0.4 or 0.8 g/min.) into the heated barrel (63**–**60 °C) and extruded through a 1 mm-die, screw speed was 150 rpm.

Formulation	Protein	Components (%)		Preparation method	Process parameters
		Protein	PEG 20,000		
Lysozyme reference	LYS	100	0	–	
Physical mixture	LYS	40	60	Turbula mixer	10 min, 50 rpm
LR40	LYS	40	60	Ram extrusion	3 min, 63 °C, 1 mm/s, 1 mm
LC40	LYS	40	60	TSE (conveying)	0.4 g/min., 150 rpm, 63–60 °C, 1 mm
LK40	LYS	40	60	TSE (kneading)	
BSA reference	BSA	100	0	–	
Physical mixture	BSA	40	60	Turbula mixer	10 min, 50 rpm
BR40	BSA	40	60	Ram extrusion	3 min, 63 °C, 1 mm/s, 1 mm
BC40	BSA	40	60	TSE (conveying)	0.8 g/min., 150 rpm, 63–60 °C, 1 mm
BK40	BSA	40	60	TSE (kneading)	
Human insulin reference	IH	100	0	–	
Physical mixture	IH	40	60	Turbula mixer	10 min, 50 rpm
IHR40	IH	40	60	Ram extrusion	3 min, 63 °C, 1 mm/s, 1 mm
IHC40	IH	40	60	TSE (conveying)	0.4 g/min., 150 rpm, 63–60 °C, 1 mm
IHK40	IH	40	60	TSE (kneading)

### 2.3 Chemical stability and protein concentration in extrudates

Chemical stability and protein concentration in extrudates were determined by HPLC (Agilent 1100 Series equipped with, quaternary pump, diode array and multiple wavelength detector (DAD), Agilent Technologies, Waldbronn, Germany)) using a C18 reversed phase column (NUCLEOSIL 120–3, C18, 5 μm, 125 × 4 mm). Protein-loaded PEG-extrudates were dissolved in Milli-Q® water and properly mixed for the extraction of protein (*n* = 3). 1.5 mL of the supernatant was used for analysis. The solvent system consisted of water/acetonitrile/trifluoroacetic acid (A: 100/0/0.1, B: 0/100/0.1, *V*/V). A linear gradient method was applied (0–5-7 min 5–95-5%B) at a flow rate of 1 mL/min for 7 min and a column temperature of 30 °C. Samples (10 μL) were injected, and chromatograms obtained with an UV-detector were quantified at 280 nm.

### 2.4 Size-exclusion chromatography

SEC was performed with a Superdex 75 Increase 10/300 GL column, 10 × 300 mm (Cytiva, Marlborough, MA, USA), where the mobile phase was 50 mM pH 7.0 phosphate buffer containing 400 mM sodium chloride at a flow rate of 0.8 mL/min and a column temperature of 30 °C. Samples were prepared as triplicates by dissolving the protein powder, physical mixture, or protein-loaded extrudates in the mobile phase with a target concentration of 3 mg/mL protein. Human insulin containing samples were pre-dissolved in 0.05 M hydrochloric acid aqueous solution and then diluted with the mobile phase. The UV detector (SPD-40 UV Detector, Shimadzu, Japan) wavelength was 214 nm. Sample vials were cooled at 15 °C in the autosampler. 20 μL of each sample was injected and potential aggregate and/or fragment formation after processing was analyzed in comparison with a chromatogram of the freshly prepared protein powder and physical mixture samples.

### 2.5 Scanning electron microscopy coupled with energy-dispersive X-ray spectroscopy (SEM-EDX) and image analysis of SEM-EDX-maps

A scanning electron microscope (SU 3500, Hitachi High Technologies, Krefeld, Germany), equipped with a backscattered electron detector (BSE) was used to investigate the morphology of the surfaces and cross sections of the prepared protein-loaded extrudates. BSE images were collected at an acceleration voltage of 5 kV at a variable pressure mode of 30 Pa. The cut extrudates were placed on an aluminum stub and sputtered with a thin platinum layer (Sputter Coater, Cressington Scientific Instruments, Watford, England). The elemental distributions were investigated by SEM combined with an energy-dispersive X-ray detector (EDX) (EDAX Element-C2B, Ametek, Weiterstadt, Germany). Protein distribution was examined by elemental mapping of the cross sections of extrudates for the characteristic X-ray peak of nitrogen. The percentage of detected nitrogen was evaluated by the TEAM software (Version 4.4.1, Ametek, Weiterstadt, Germany). Image analysis of SEM/EDX-maps was used to parameterize the distribution of the protein particles in the PEG-matrix. The percentage of the area with protein was calculated using image editing software ImageJ (ImageJ 1.53q, National Institutes of Health, USA). The coefficient of variation (CV) of the average protein particle area (nitrogen signal in green) was calculated. This relative protein particle area variation (calculated as CV in %) was taken as a descriptor for the protein particle distribution. Low levels in CV indicate a homogenous protein particle distribution, whereas high levels would represent a high variation of protein particle distribution, i.e., an inhomogenous particle distribution (Steffens et al., 2020). For every formulation, three extrudate cross sections (Ø = 1 mm) were analyzed in total for mean value and standard deviation calculation of this distribution value.

### 2.6 Solid-state differential scanning calorimetry (ss-DSC)

DSC studies of protein powder, physical mixture and extrudates were performed with a DSC 2 (Mettler Toledo, Gießen, Germany) equipped with an auto sampler, nitrogen cooling and nitrogen as purge gas (30 mL/min). The system was calibrated with indium and zinc standards. At least three samples of ∼10 mg were accurately weighed in 40 μL aluminum crucibles with a pierced lid. DSC scans were recorded from 25 °C to 230 °C using a heating rate of 10 K/min. STAR^e^ software (Mettler Toledo, Gießen, Germany) was employed for acquiring thermograms. Thermograms were normalized for sample weight and the peak minimum was designated as unfolding temperature of the protein.

### 2.7 Solid-state Fourier Transform infrared spectroscopy (ss-FTIR)

ss-FTIR spectra were generated with a Spectrum Two™ FT-IR spectrophotometer equipped with an UATR accessory (PerkinElmer Inc., Waltham, USA). The measured samples included protein powders, physical mixtures and extrudates. Protein-loaded extrudates were milled with a mortar and pestle prior measurement. A tight pressure clamp with a flat tip ensured a good contact between the sample and the reflection diamond crystal. Each sample was measured as triplicate. The spectra were recorded against an air background between 4000 and 400 cm^−1^ with an average of four scans and a resolution of 4 cm^−1^. Data were collected in the absorption mode. First and second derivative analysis was performed with GraphPad PRISM Software (San Diego, CA, USA). The secondary structure elements (i.e., β-sheet structure, α-helix, β-turn, or unordered structures) were considered in the amide-I region (1700–1600 cm^−1^) of the protein.

### 2.8 Circular-dichroism spectroscopy

In order to analyze the secondary structure of the proteins, Far-UV-CD was exploited. Firstly, samples of pure proteins serving as reference samples were diluted to a concentration of 0.4 mg/mL in pure water (BSA and Lysozyme) or 0.5% formic acid in pure water (Insulin human) as insulin turned out to be only poorly soluble at a neutral pH. Following this, 40% protein extrudates were diluted to concentrations ranging from 0.4 mg/mL to 1.0 mg/mL of the respective extrudate (0.16 mg/mL protein to 0.4 mg/mL protein) with pure water (BSA and Lysozyme samples) or with 0.5% (*v*/v) formic acid in pure water (human insulin samples). In addition, a solution containing 0.6 mg/mL PEG 20,000, resembling the PEG 20,000 concentrations from the protein extrudate samples was prepared in pure water and in 0.5% formic acid in pure water. The CD spectra were recorded using a spectropolarimeter (J-715, Jasco Corp., Japan) by scanning the sample from 195 to 260 nm at 20 °C using a 0.1 cm quartz cuvette. Bandwidth, sensitivity, and scanning speed was set at 0.5 or 1.0 nm, 100 mdeg and 100 nm/min, respectively. Three spectra were recorded with each spectrum being the average of three scans. Initially, datasets were recorded of the respective solvents lacking of the respective protein component and of the PEG only containing solutions. They were used for subtraction of the solvent, respectively PEG 20,000 background from the actual protein containing spectra in the Jasco spectra manager software. After subtraction, these spectra were smoothed using the preset values in the Jasco spectra manager software.

### 2.9 Biological activity of Lysozyme

Lysozyme activity was determined by a fluorescence-based assay (EnzChek® Lysozyme Assay Kit, Molecular Probes Europe BV, Leiden, The Netherlands) using a suspension of *Micrococcus lysodeikticus* labeled with fluorescein. The assay determines the Lysozyme activity on labeled cell walls of *Micrococcus lysodeikticus*. The fluorescence increase was measured using a microplate reader with a fluorescein filter and OptiPlate™-96 F microwell plates (VICTOR3™ Multilabel Plate Reader; 96-Well plates black, PerkinElmer Life and Analytical Sciences, Shelton, USA). Preparation of DQ Lysozyme substrate stock suspension, Lysozyme standard curve, as well as the procedure were conducted according to the manufacturer's protocol. The reaction mixtures were incubated at 37 °C for 30 min, protected from light. The fluorescence intensity of each reaction in a microwell plate was measured at 494 nm (absorption maximum) and 518 nm (emission maximum). The fluorescence values obtained from the control without enzyme were subtracted.

### 2.10 Accelerated thermal stress test

To assess the protein stability under thermal stress (accelerated conditions) protein-loaded PEG-extrudates and protein powder (reference) were stored at 40 °C for 28 days. Briefly, protein-loaded PEG-extrudates and protein powder (Lysozyme, BSA, and human insulin) were stored in an upright orientation (Eppendorf LoBind, 0.5 mL, Eppendorf, Hamburg, Germany) at 40 ± 1 °C (Thermocabinet BE400, Memmert GmbH & Co. KG, Schwabach, Germany) for 28 days. After 28 days of storage, the samples were analyzed by (i) RP HPLC (chemical stability and protein concentration), (ii) SEC (protein fragment and aggregation analysis), (iii) DSC (protein's unfolding temperature), (iv) FTIR spectroscopy (conformational stability), and (v) activity assay (only for Lysozyme).

### 2.11 Statistical analysis

Statistical analysis and testing for statistical significance was carried out using GraphPad PRISM Software (San Diego, CA, USA).

## 3 Results & discussion

During melt extrusion mainly the stress factors heat exposure in combination with shear stress, are potentially affecting protein stability and consequently can lead to their degradation or inactivation. Within the current study, the analytical investigation and characterization of protein stability in highly-loaded protein extrudates was demonstrated. The stability of Lysozyme, BSA, and human insulin after ram extrusion and TSE at low temperatures (<70 °C) was evaluated by RP-HPLC (chemical stability and protein concentration), SEC (protein fragment and aggregation analysis), SEM-EDX (protein particle distribution over extrudate cross section), DSC (unfolding temperature of the proteins), CD and FTIR spectroscopy (conformational stability), and in case of Lysozyme biological activity (Lysozyme activity assay).

### 3.1 Chemical protein stability and protein concentration in extrudates

**Fig. 3:**
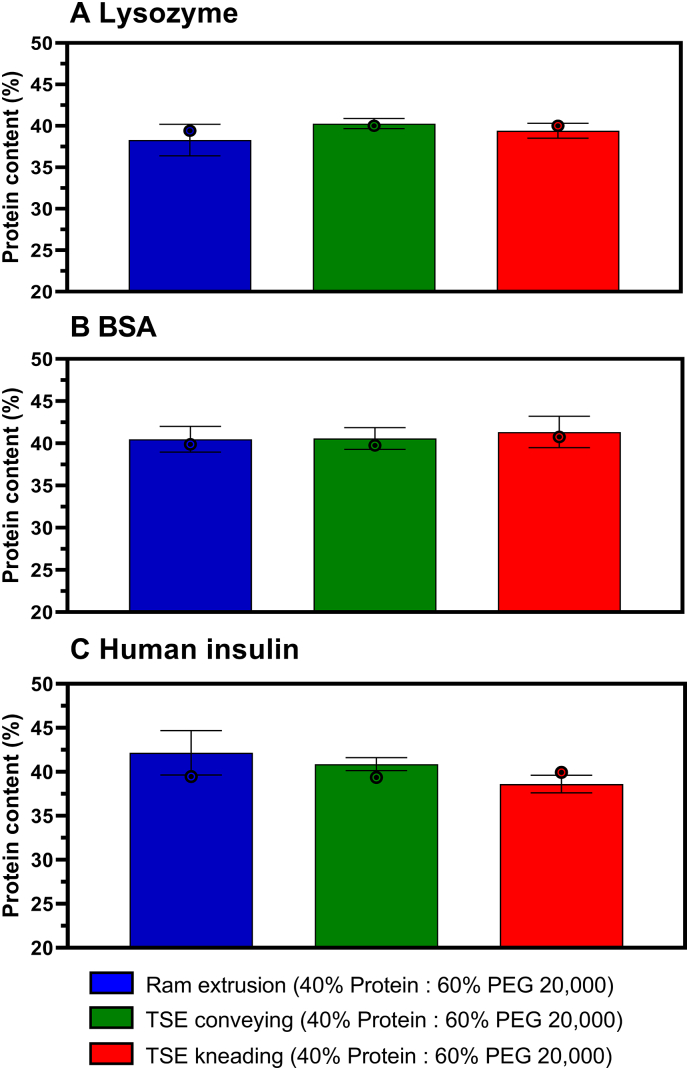
Protein concentration in 40% protein-loaded extrudates (*n* = 3) prepared by ram extrusion (blue) or TSE with only conveying screw configuration (green), or screws containing a single 90° kneading element (red), at 63 °C; A Lysozyme-loaded extrudates, B BSA-loaded extrudates, and C human insulin-loaded extrudates; bars represent the measured protein concentration; error bars represent the standard deviation of three measurements for the protein content by RP-HPLC; the dots show the target protein concentration based on the protein concentration of the processed physical mixture. (For interpretation of the references to colour in this figure legend, the reader is referred to the web version of this article.)

Chemical degradation of a protein due to oxidation can occur during any stage of protein production, purification, formulation and storage and any protein that contains Histidine, Methionine, Cysteine, Tyrosine and Tryptophan amino acids is susceptible to oxidation (Manning et al., 2010). The degradation behavior (i.e., appearance of chemically and structurally modified degradation products) of Lysozyme, BSA, and human insulin after melt extrusion was monitored by RP-HPLC. Chemical stability analysis of the extracted proteins by RP-HPLC showed that no oxidation or hydrolysis of the proteins occurred during ram extrusion or TSE at 63 °C. The protein content in extrudates was also analyzed by RP-HPLC and compared to the theoretical or target protein concentration (Fig. 3). The theoretical protein concentration resulted from the weighed amounts (40% protein powder and 60% PEG 20,000) of the components to prepare the respective physical mixture. The actual protein concentration was measured by RP-HPLC as mentioned before. The protein concentration in ram extrudates showed the highest deviation of target and actual protein concentration (Δc = difference between target and actual concentration) compared to extrudates prepared by TSE and was 1.9, 1.5, and 2.5% for Lysozyme-, BSA-, and human insulin-loaded extrudates, respectively. TSE with conveying screw configuration showed the lowest deviation in protein content Δc = 0.3, 0.8, and 1.50% for Lysozyme, BSA, and human insulin, respectively) and hit the target concentration closest. The results also highlight, that the use of conveying screws was satisfactory in terms of homogenous protein particle distribution and the use of a single kneading element did not improve the distribution and was thus not superior. In contrast to ram extrusion, TSE uses co-rotating screws to press the molten material through a cylindrical die. Once the physical powder mixture has passed through the feed system to the screws, it is conveyed by the turning motion of the screws along the extruder barrel, which introduces mixing and heat into the material through both external heaters and friction between the screws, barrel and within the material (viscous dissipation).

### 3.2 Fragment and protein aggregation analysis

Protein denaturation can occur and lead to aggregation and formation of high molecular weight species (HMWS). When the protein is denatured (partially or completely), more hydrophobic parts of the molecule are exposed, as well as a higher flexibility of the whole protein molecule (Wang et al., 2010). However, proteins in solid-state formulations can tend to aggregate without any detectable change in secondary structure and are therefore indistinguishable from native protein species using techniques such as FTIR spectroscopy (Griebenow and Klibanov, 1995). SEC is a commonly used method for the detection of protein aggregation and fragment formation. The aggregation behavior of Lysozyme, BSA, and human insulin after melt extrusion was monitored and HMWS or fragments were separated from monomeric derivatives. SEC revealed, that extrusion did not trigger the formation of protein fragments or protein aggregates in the case of Lysozyme and BSA. Lysozyme was only present as a monomer after dissolving the extrudates in eluent. Native BSA occurred as a mixture of monomers and dimers, however the ratio of these species was not negatively affected by ram extrusion or TSE. Native human insulin appeared as a species at a retention time of 21 min. A second small peak in front of the peak of native human insulin appeared in combination with PEG 20,000. This second population indicated the formation of HMWS in the presence of PEG 20,000. However, the amount of HMWS was <0.2% compared to the monomer species of human insulin. Furthermore, the formation of the HMWS was independent of the extrusion process, since HMWS appeared also in the case of the unprocessed, physical mixture composed of 40% human insulin and 60% PEG 20,000. This observation highlighted that human insulin molecules interacted with hydrophilic PEG 20,000 molecules may be due to a PEG-induced alteration of the local peptide structure. Recent studies suggest that stabilization of a protein by PEG is dependent on the protein size and its sequence and is thus not generally transferable (Chao et al., 2014). As these interactions are unfavorable, we cannot recommend PEG 20,000 as polymeric matrix and protein stabilizer for human insulin formulations.

### 3.3 Protein particle distribution over the cross sections of extrudates

**Fig. 4:**
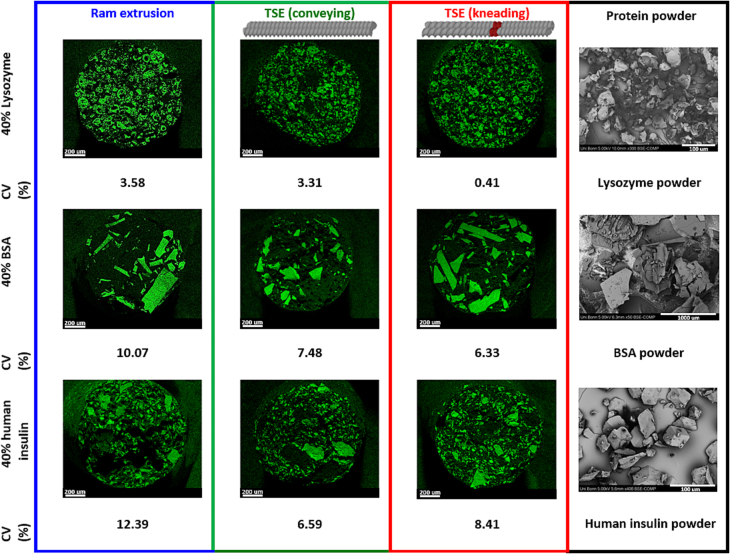
Protein particle distribution of Lysozyme, BSA, human insulin illustrated with EDX-SEM elemental mapping of nitrogen (green spots) on cross-section of 40% protein-loaded extrudates prepared by ram (blue) or TSE with only conveying screw configuration (green), or screws containing a single 90° kneading element (red), at 63 °C; and coefficients of variation (%) of the image analyses of SEM-EDX-maps. The scale bar corresponds to 200 μm. SEM-images of the used protein powder (black) are shown on the right column. (For interpretation of the references to colour in this figure legend, the reader is referred to the web version of this article.)

Protein particle distribution was analyzed by SEM-EDX. Images of the cross section of extrudates produced at 63 °C using ram extrusion or TSE were compared. The cross-section cut of the extrudates prepared by ram extrusion (Fig. 4) showed no pores or cracks inside of the extrudates, whereas the extrudates prepared by TSE showed only few pores (Fig. 4). Elemental mapping of the protein's nitrogen atoms showed a homogenous distribution of protein particles in extrudates prepared by ram extrusion and TSE at 63 °C. Furthermore, the protein particles were solely embedded in the hydrophilic matrix of PEG 20,000 and not shredded or dissolved during ram extrusion or TSE (Fig. 4). Moseson et al. (2022) discus the dependence of the residence time during hot melt extrusion and the dissolution rate of drug crystals in the molten polymer. The crystal model drugs showed a significant residual crystallinity for short residence times (2 min.), indicating a low crystal dissolution rate (Moseson et al., 2022). During our TSE experiments, the residence times were < 1 min assuming that the time for dissolving and dissolution rate of protein powder particles in molten PEG 20,000 was extremely small. The shown particle morphology of embedded protein particles compared to the used protein powder particles (right column of Fig. 4) confirmed the hypothesis of a solely embedment in the hydrophilic matrix of PEG 20,000 without dissolving or recrystallization of the protein particles during melt extrusion processing. The protein particles probably act as filler material resulting in a reduced amount of energy required for the viscous dissipation during melt extrusion processing. Coefficients of variance were used to describe the protein particle distribution over the cross section of extrudates quantitatively. Protein-loaded extrudates prepared by ram extrusion showed the highest CV (3.58, 10.07, and 12.39% for Lysozyme, BSA, and human insulin, respectively) compared to extrudates processed by TSE (conveying screw configuration: 3.31, 7.48, and 6.59%; kneading screw configuration: 0.41, 6.33, and 8.41% for Lysozyme, BSA, and human insulin, respectively) and is thus associated with a less homogenous particle distribution. During ram extrusion the molten blend is forced through the die with the aid of a driving piston. The barrel of the ram extruder does not contain any restrictive elements for mixing like the co-rotating screws of a twin-screw extruder. However, the homogeneity depends on the initial particle size and distribution of the protein particles. In general, the small lysozyme particles resulted in low CV at all processing variants. Ram extrusion provided also homogenously distributed protein-loaded extrudates when small-sized protein particles (<200 μm) with a narrow particle distribution will be used. In contrast to TSE, ram extrusion offers the potential to prepare highly-loaded solid protein formulations at high yield, high drug load and at low shear stress.

### 3.4 Unfolding temperature

**Fig. 5:**
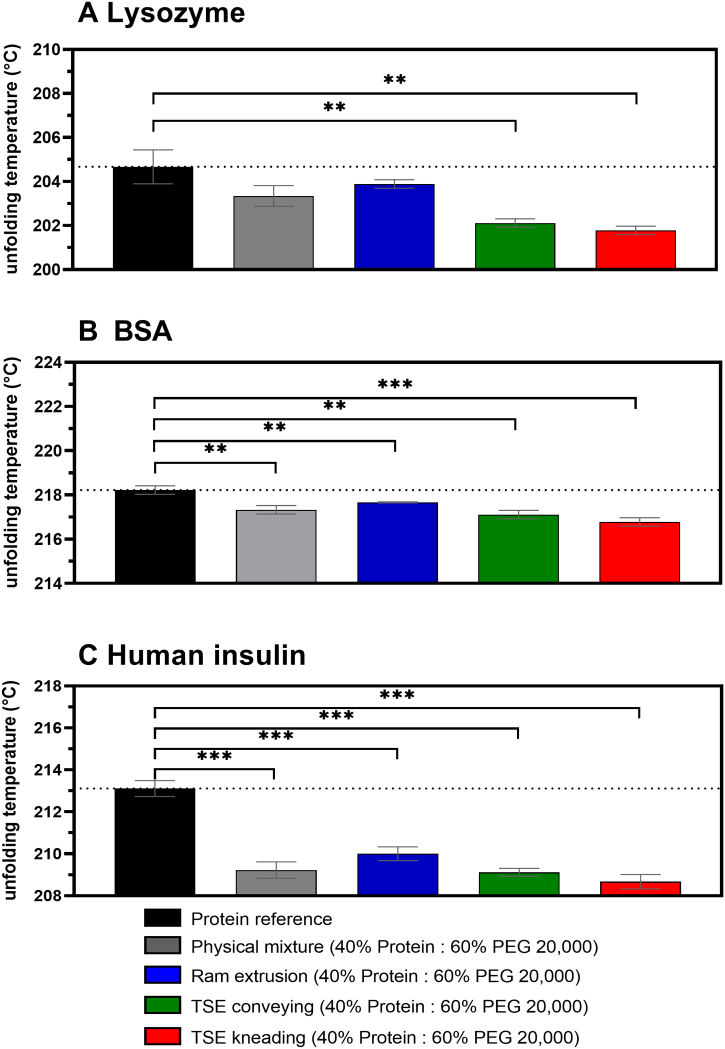
Unfolding temperature of protein powders (black), physical mixtures (gray), and 40% protein-loaded extrudates prepared by ram extrusion (blue) or TSE with only conveying screw configuration (green), or screws containing a single 90° kneading element (red), at 63 °C; A Lysozyme-loaded extrudates, B BSA-loaded extrudates, and C human insulin-loaded extrudates; the dotted line shows the unfolding temperature of the unprocessed protein (reference). Error bars represent the standard deviation of three measurements for the unfolding temperature by DSC; unpaired *t*-test (two-sample assuming equal variances) was used and statistical significance was depicted by asterisks (*): * *p* < 0.05, ** *p* < 0.01, *** *p* < 0.001, **** *p* < 0.0001. (For interpretation of the references to colour in this figure legend, the reader is referred to the web version of this article.)

The globular structure of proteins is referred to as the native state. Unfolding and denaturation is mostly accompanied by a loss of that globular structure or the loss of secondary or tertiary structures that most proteins adopt. Consequently, upon unfolding or denaturation, the protein structure changes its physical state, but maintaining the chemical composition (Manning et al., 2010). Proteins do not melt, but they change their molecular conformation from a native to a denatured state at the unfolding temperature. DSC was used to measure the protein unfolding temperature in solid-state (midpoint being denoted as the T_m_ value) (Ghalanbor et al., 2010). At T_m_ the attractive intra-molecular forces were overcome, which preserve their native state. The reported denaturation temperatures for proteins in the solid-state are predominately high and often above 200 °C (Ghalanbor et al., 2010). DSC thermograms exhibited broad peaks designated as unfolding temperatures (T_m_) of 204.7 °C, 218.2 °C, and 213.1 °C for native Lysozyme, BSA, and human insulin, respectively (Fig. 5). Moreover, Fig. 5 shows that the unprocessed proteins unfolded and a peak was detected at their apparent denaturation temperatures, which varied according to the protein (Ghalanbor et al., 2010; Mohammad et al., 2015; Elkordy et al., 2002; Mohammad, 2016; Han et al., 2012). In general, an increasing unfolding temperature reflects an increase in conformational protein stability. The physical mixtures composed of 40% protein powder and 60% PEG 20,000, showed reduced unfolding temperatures for all the used proteins compared to the reference samples may be due to the formation of an eutectic mixture. This observation was confirmed by DSC scans of physical mixtures composed of 20, 40, and 60% BSA (Supplementary Information, Fig. S1). The unfolding temperature of BSA was reduced in the presence of PEG 20,000, and depending on the amount of polymer (80, 60, and 40% PEG 20,000, ranking order: 216.3, 217.3, and 218.6 °C, respectively). Presumably the most common stress that can cause a loss of protein structure is elevated temperature and designated as thermally induced protein denaturation (unfolding). The presence of a melting peak >200 °C in the DSC scan can indicate conservation of protein conformation after extrusion at 63 °C. Lysozyme showed a significantly reduced unfolding temperature after TSE with conveying screws (202.1 °C) and a screw configuration containing a single kneading element (201.8 °C) at 63 °C. The ram extrusion process did not affect the unfolding temperature (203.9 °C) of Lysozyme significantly. For BSA and human insulin the applied formulation processes significantly affected the unfolding temperature towards lower temperatures compared to the reference samples. However, the total reduction of unfolding temperatures compared to the reference samples were < 1.5 °C and < 4.5 °C for BSA- and human insulin-containing extrudates, respectively. DSC was also used to evaluate extrusion processing and effects of the used extrusion process on the thermal stability of proteins. Ram extrusion resulted in the lowest, whereas TSE with a kneading screw element led to the largest reduction of unfolding temperatures of Lysozyme, BSA, and human insulin. The thermal unfolding mechanisms of Lysozyme, BSA, and human insulin include the appearance of a single melting peak. The proteins exhibit an irreversible unfolding mechanism (Mohammad, 2016) as was seen in the second DSC scan, since the single peak of the first scan was completely disappeared (data not shown).

### 3.5 Conformational stability and secondary structure analysis

**Fig. 6:**
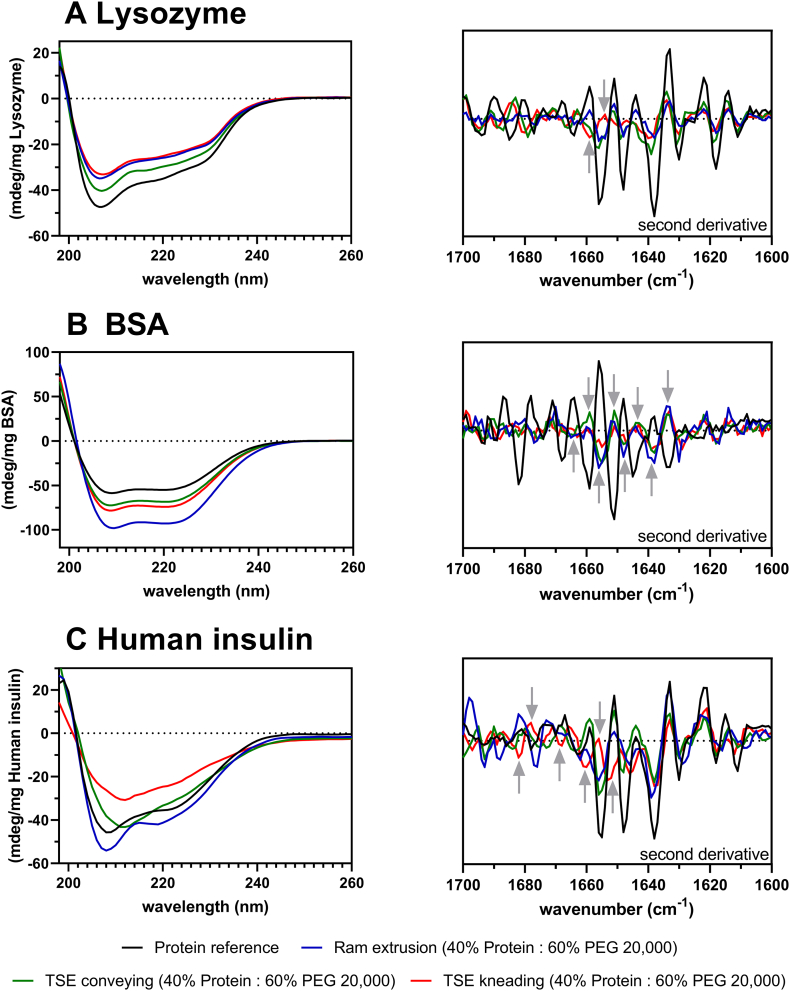
Left: CD spectra of A Lysozyme, B BSA, and C human insulin (protein reference samples in black) in aqueous solution and 40% protein-loaded extrudates after reconstitution at 20 °C; initial ellipticity values were corrected by the respective amount of protein weighed in for the measured solution. Right: Second derivative of FTIR spectra in the Amide *I* region (1600–1700 cm^−1^) of protein powder (black), and 40% protein-loaded extrudates prepared by ram extrusion (blue) or TSE with only conveying screw configuration (green), or screws containing a single 90° kneading element (red), at 63 °C; gray arrows highlight changes in secondary structure elements. (For interpretation of the references to colour in this figure legend, the reader is referred to the web version of this article.)

CD and FTIR spectroscopy were used to compare the molecular conformation and secondary structure elements of protein powder before and after melt extrusion. The secondary structure information of powdered Lysozyme, BSA, and human insulin was used as a reference (Fig. 6). The model protein Lysozyme is a small globular protein having a molecular weight of 14.4 kDa, five helical regions, five regions of beta sheet, a large amount of random coil and beta turns (Al-Azzam et al., 2002). The secondary structure elements of Lysozyme as obtained from the Protein Data Bank mainly include α-helices (34%–42%) and β-sheets (7%–12%) (Sheng et al., 2016; White, 1976). The native Lysozyme spectrum showed two minima around 206 and 220 nm, representing the main features of α-helical secondary structure. The conformational stability of Lysozyme in PEG-extrudates was confirmed by CD and FTIR spectroscopy. The conformational stability of Lysozyme was not negatively affected by ram extrusion or TSE. The spectra of extrudates containing 40% Lysozyme and prepared by ram extrusion or TSE were comparable to the native Lysozyme powder used to prepare the physical mixture and thus the extrudates (Fig. 6A). There was also no indication for denaturation (shifts or distortion of bands) or aggregation (intermolecular β-sheet formation) as a consequence of the heat exposure and shear stress during ram extrusion or TSE at 63 °C. Previous studies using CD and FTIR spectroscopy have reported the secondary structure of BSA (M_w_ = 66.5 kDa). These studies showed that the α-helical content of BSA ranges from 52% to 68% also containing 10% turns, but no β-sheets (Chen et al., 2021b; Servagent-Noinville et al., 2000; Lu et al., 2015). The CD spectra of all BSA samples were mostly overlapping, however in the FTIR spectra some shifts are shown. A probable explanation for the observed differences in the FTIR spectra at 1652 cm^−1^ may be the extrusion procedure, since extrusion (thermal and shear stress) can cause reversible changes in protein conformation and is associated with a reduction in the α-helical content and an increase in the random chain content (Bolje and Gobec, 2021; Bagińska et al., 2008). Human insulin was also studied for changes in its secondary structure as a result of temperature and shear stress during extrusion processing. Human insulin (M_w_ = 5.8 kDa) is a peptide and composed of two peptide chains (A- and B-chain) including three α-helices and a short β-sheet segment (Fig. 6B) (Ciszak et al., 1995). CD spectra of human insulin-loaded extrudates prepared by TSE showed clear shifts and changes in secondary structure elements. The shifts were also confirmed by the FTIR spectra (second derivative). The second derivative spectra of the Amide *I* band suggested a red-shift of the bands around 1650–1665 cm^−1^, assigned to turns and β-sheet, which can be caused by small loosening of the turn structures. These changes indicated the transformation of α-helix content to β-sheets or a disordered structure which enhances the tendency to aggregate. In particular, the band at 1652 cm^−1^ which corresponds to α-helical structures (Bolje and Gobec, 2021), was obviously affected by TSE with screws containing a kneading element due to higher shear stress and residence time. As can be seen in the CD and FTIR spectra, ram extrusion had the lowest impact on secondary structure changes of human insulin (Fig. 6C).

### 3.6 Protein stability after accelerated thermal stress

An accelerated thermal stability study was applied to obtain protein stability data. At the end of the storage period, the samples (unstressed protein powder as reference, protein powder and protein-loaded extrudates stored at 40 °C for 28 days) were analyzed by HPLC, SEC, DSC, and FTIR spectroscopy. Lysozyme containing PEG-extrudates were additionally tested for a potential loss of biological activity (refer to Section 3.7).

**Fig. 7:**
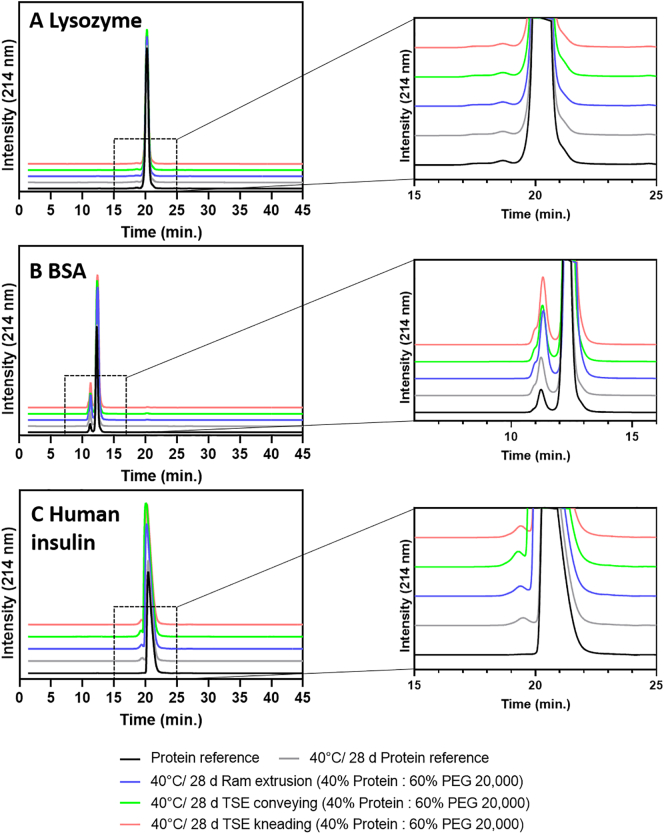
Chromatograms of SEC analysis after an accelerated stability study at 40 °C for 28 days. Protein powders (initial: black, after 28 days at 40 °C: gray), and 40% protein-loaded extrudates prepared by ram extrusion (blue) or TSE with only conveying screw configuration (green), or screws containing a single 90° kneading element (red), at 63 °C; A Lysozyme-loaded extrudates, B BSA-loaded extrudates, and C human insulin-loaded extrudates. Right: Magnified areas of the peak of interest. (For interpretation of the references to colour in this figure legend, the reader is referred to the web version of this article.)

Chemical degradation of the protein after storage at 40 °C was investigated by HPLC. The results showed that no oxidation or hydrolysis of the proteins occurred after storage. Protein denaturation, protein aggregation or formation of HMWS are also potential events occurring during thermal storage of protein formulations. The aggregation behavior of Lysozyme, BSA, and human insulin after storage was monitored and HMWS or fragments were separated from monomeric derivatives by SEC. Results showed, that the thermal storage of Lysozyme-loaded extrudates did not result in the formation of protein aggregates. Lysozyme was only present as a monomer after dissolving the extrudates in eluent (Fig. 7A). Native BSA occurred as a mixture of monomers and dimers (Fig. 7B). The BSA-containing samples (i.e., BSA powder, and BSA-loaded extrudates) stored at 40 °C showed a small shoulder of the dimeric peak assuming that trimers were present in the samples. BSA trimers occurred also in the stored BSA powder and thus the impact of melt extrusion processing was not the driver for the formation of trimers. Native human insulin showed a single peak at a retention time of 21 min. (Fig. 7C). A second small peak in front of the peak of native human insulin appeared after storage at 40 °C. This second population indicated the formation of HMWS due to thermal stress during storage over 28 days. However, the amount of formed HMWS was in the same magnitude as the stored unprocessed human insulin powder.

**Fig. 8:**
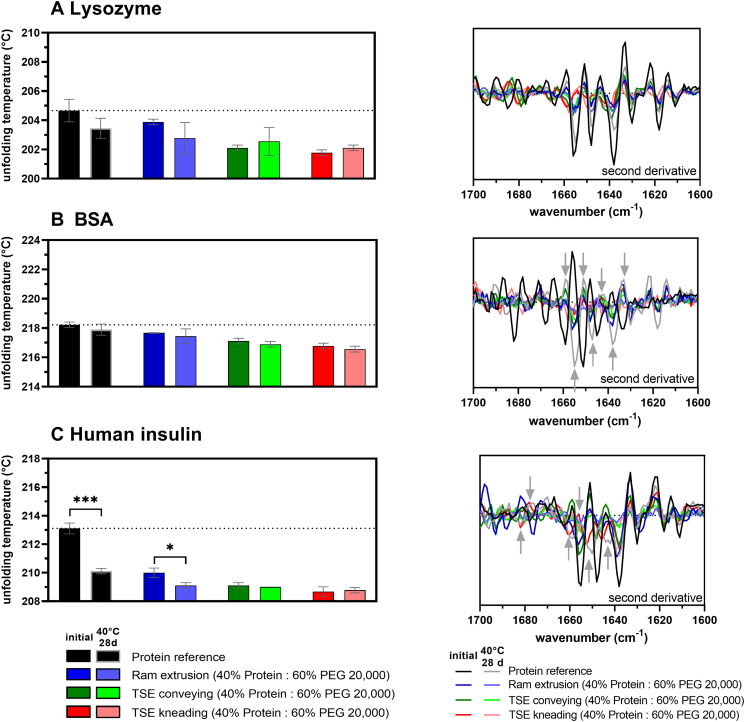
Results of accelerated thermal stability study at 40 °C for 28 days. Left: Unfolding temperature of protein powder references (black), and 40% protein-loaded extrudates prepared by ram extrusion (blue) or TSE with only conveying screw configuration (green), or screws containing a single 90° kneading element (red), at 63 °C determined by DSC; A Lysozyme-loaded extrudates, B BSA-loaded extrudates, and C human insulin-loaded extrudates; the dotted line shows the melting unfolding temperature of the unprocessed protein (reference). Bars with gray border colour represents the unfolding temperatures after an accelerated storage stability study at 40 °C for 28 days. Error bars represent the standard deviation of three measurements for the melting unfolding temperature by DSC; unpaired *t*-test (two-sample assuming equal variances) was used and statistical significance was depicted by asterisks (*): * *p* < 0.05, ** *p* < 0.01, *** *p* < 0.001, **** *p* < 0.0001. Right: Second derivative of FTIR spectra in the Amide *I* region (1600–1700 cm^−1^) of protein powder reference (black and gray), and 40% protein-loaded extrudates prepared by ram extrusion (blue) or TSE with only conveying screw configuration (green), or screws containing a single 90° kneading element (red), at 63 °C; gray arrows highlight changes in secondary structure elements. (For interpretation of the references to colour in this figure legend, the reader is referred to the web version of this article.)

Protein unfolding and denaturation is mostly accompanied by a loss of the specific globular protein structure or the loss of secondary or tertiary structure elements. DSC was used to measure the protein unfolding temperature in solid-state (Fig. 8). In the case of Lysozyme, the unfolding temperature as well as the secondary structure elements were not negatively affected or shifted due to thermal stress (i.e., 28 days at 40 °C) (Fig. 8A). The unfolding temperature of BSA samples was also not significantly reduced. The FTIR spectra (second derivative) of the unprocessed, stored BSA powder showed shifts in secondary structure elements. The shifts in the amide I region of BSA-loaded extrudates were comparable to the unprocessed, stored BSA powder, indicating that melt extrusion processing did not cause these modifications. The stability of the unprocessed and stored human insulin powder was significantly affected by thermal stress (Fig. 8C) resulting in a reduced unfolding temperature and shifts in secondary structure elements compared to the reference. The secondary structure and unfolding temperature of human insulin was less affected in extrudates produced by ram extrusion or TSE during storage at 40 °C.

### 3.7 Biological Lysozyme activity after extrusion and accelerated thermal stress

**Fig. 9:**
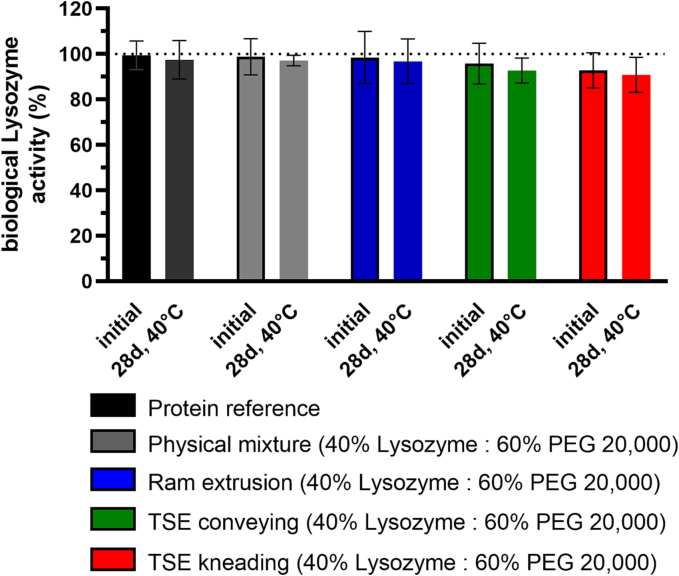
Biological activity of Lysozyme powder (black), the physical mixture (gray), and 40% Lysozyme-loaded extrudates prepared by ram extrusion (blue) or TSE with only conveying screw configuration (green), or screws containing a single 90° kneading element (red), at 63 °C; initial: samples were immediately analyzed after melt extrusion processing; Bars without border represent the biological activity of Lysozyme of samples stored at 40 °C for 28 days; Error bars represent the standard deviation of three measurements. (For interpretation of the references to colour in this figure legend, the reader is referred to the web version of this article.)

Therapeutic proteins can rapidly denature and lose their function due to applied stresses during processing. Despite Lysozyme is known as one of the most stable proteins, it is an accepted model for investigating the relationship between protein structure and biological function. I.e., if a process results in a significant loss in activity of Lysozyme this process would disqualify itself for processing of other (model) proteins. The biological activity of Lysozyme was investigated immediately after ram extrusion and TSE at 63 °C and an accelerated thermal stress test at 40 °C for 28 days (refer to Section 3.6). Lysozyme embedded in melt extrudates by ram extrusion or TSE at 63 °C and after the accelerated stress test maintained full enzymatic activity when compared to an unprocessed or initial sample (*t*-test: *p* < 0.05) as expected (Fig. 9). The assay showed a satisfactory activity of Lysozyme after ram extrusion and TSE and highlights the extreme stability of Lysozyme against heat exposure and shear stress. This observation was confirmed by the results of other applied analytical methods in our study (i.e., RP-HPLC, SEC, DSC, CD and FTIR spectroscopy) and a prior conducted SDS-PAGE analysis of the Lysozyme-loaded extrudates by (Elsayed et al., 2022). Here, the Lysozyme-loaded PEG-extrudates analyzed by one dimensional SDS-PAGE at reducing and nonreducing conditions showed always a clear single band for the samples between 10 and 15 kDa (∼14 kDa) without indication of impurities or degradation products of Lysozyme after melt extrusion processing.

## 4 Conclusion

In this study, we addressed the investigation of highly-loaded protein PEG-extrudates prepared by ram extrusion and TSE with regard to the main challenges, i.e., protein instability due to heat exposure and shear stress during extrusion. The applied methods, including (i) RP-HPLC (chemical stability and protein concentration), (ii) SEC (protein fragment and aggregation analysis), (iii) SEM-EDX (protein particle distribution), (iv) DSC (protein's unfolding temperature), (v) FTIR and CD spectroscopy (conformational stability), and (vi) activity assay, enabled the successful characterization of protein stability in extrudates (mg-scale) and proved to be very useful techniques to study process-related effects on protein stability and changes in model proteins induced by melt extrusion processing. PEG 20,000 was introduced as a first and simple polymer model due to its low processing temperature (< 70 °C) and hydrophilicity (i.e., simplifying the sample preparation and analytical investigations of protein-loaded extrudates). This study also offers the potential to compare different melt extrusion processes (i.e., ram extrusion vs. TSE (conveying) vs. TSE (kneading)) and extrusion parameters (e.g., shear stress levels, screw configurations, residence times). PEG 20,000 was used as a model polymer to embed and stabilize the proteins by interacting with the protein to decelerate the unfolding rate of the embed proteins. We showed the successful and homogenous embedment of proteins in PEG 20,000 matrices without dissolution of the protein particles in the polymer for solid-state stabilization. Nearly complete recovery of active Lysozyme illustrated that ram extrusion and TSE did not negatively affect the protein integrity. However, differences seen between Lysozyme- and BSA- or human insulin-loaded extrudates indicated that melt extrusion processing could have an impact on the conformational stability. In particular, BSA and human insulin were more susceptible to shear stress compared to Lysozyme. Consequently, ram extrusion led to less conformational changes compared to TSE due to lower shear stress. The use of conveying screws in TSE was satisfactory in terms of homogenous protein particle distribution and the use of a single kneading element did not improve the distribution. Ram extrusion showed also a good protein particle distribution and is especially for small-sized protein particles (<200 μm) the preferred method to prepare highly-loaded solid protein formulations (i.e., satisfactory protein particle distribution accompanied with lower shear stress compared to TSE). As the results showed unfavorable interactions between PEG chains and human insulin molecules, the hydrophilic PEG is not the appropriate polymeric matrix for embedding human insulin and the stabilizing potential of PEG did not apply in this case. This study proposes the implementation of a sensitive characterization pathway of protein stability and investigation of the impact of process-related stress on protein stability in highly-loaded solid protein/PEG formulations from small-scale melt extrusion. Melt extrusion processing for solid-state embedment and stabilization of Lysozyme, BSA, and human insulin in PEG-matrices was neither superior nor inferior immediately after processing or an accelerated storage stability study. However, the study highlighted that melt extrusion processing offers the potential for the production of polymer-based protein embeddings in general, e.g., sustained protein release formulations. The applied workflow provides a fundamental basis for future work, including the evaluation of the use of other process techniques (e.g., vacuum compression molding or a combination of spray-drying and melt extrusion), as well as the use of sustained release polymers (e.g., PEO (release over several weeks) PLGA (release over several months, EVA (release up to 2 years) or other excipients (e.g., pH-modifier, surfactants, plasticizer).

## Research funding

This work was supported by the German Research Foundation (DFG SPP1934, project number: 273937032).

## CRediT authorship contribution statement

**Katharina Dauer:** Conceptualization, Methodology, Investigation, Data curation, Writing – original draft, Writing – review & editing, Visualization. **Christian Werner:** Investigation, Writing – review & editing. **Dirk Lindenblatt:** Investigation, Writing – review & editing. **Karl Gerhard Wagner:** Conceptualization, Resources, Writing – review & editing, Supervision.

## Declaration of Competing Interest

The authors declare no competing interests.

## Data Availability

Data will be made available on request.

## Appendix A Supplementary data

Unfolding temperatures of BSA reference and physical mixtures (60, 40, and 20% BSA and 40, 60, and 80% PEG 20,000, respectively) by DSC. **Figure S1.** Unfolding temperature of BSA reference (100% BSA) and physical mixtures (60, 40, and 20% BSA and 40, 60, and 80% PEG 20,000, respectively), the dotted line shows the melting temperature of the unprocessed protein (reference). Error bars represent the standard deviation of three measurements for the melting temperature by DSC. Unpaired t-test (two-sample assuming equal variances) was used and statistical significance was depicted by asterisks (*): * p <0.05, ** p <0.01, *** p <0.001, **** p <0.0001. Image 1
