# Supplementary material for: Impact of process stress on protein stability in highly-loaded solid protein/PEG formulations from small-scale melt extrusion
Source: Int J Pharm X. 2022 Dec 30;5:100154. doi: 10.1016/j.ijpx.2022.100154 (PMC9826855; doi:10.1016/j.ijpx.2022.100154)
Supplement: Supplementary file 1 — Unfolding temperatures of BSA reference and physical mixtures (60, 40, and 20% BSA and 40, 60, and 80% PEG 20,000, respectively) by DSC. Figure S1. Unfolding temperature of BSA reference (100% BSA) and physical mixtures (60, 40, and 20% BSA and 40, 60, and 80% PEG 20,000, respectively), the dotted line shows the melting temperature of the unprocessed protein (reference). Error bars represent the standard deviation of three measurements for the melting temperature by DSC. Unpaired t-test (two-sample assuming equal variances) was used and statistical significance was depicted by asterisks (*): * p <0.05, ** p <0.01, *** p <0.001, **** p <0.0001. [file mmc1.docx]

Supplementary Information


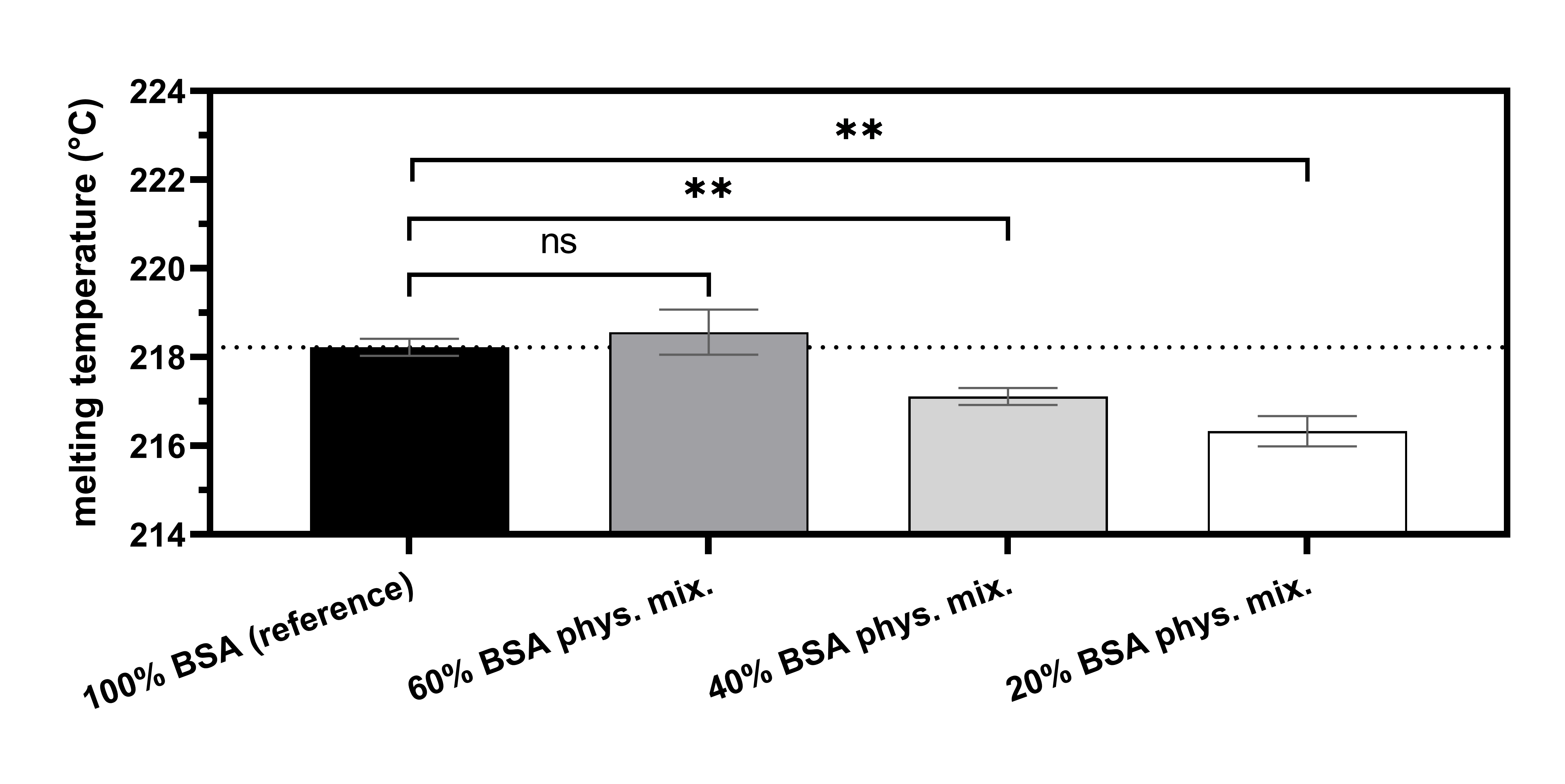


**Figure S1.** Melting temperature of BSA reference (100% BSA) and physical mixtures (60, 40, and 20% BSA and 40, 60, and 80% PEG 20,000, respectively), the dotted line shows the melting temperature of the unprocessed protein (reference). Error bars represent the standard deviation of three measurements for the melting temperature by DSC. Unpaired t-test (two-sample assuming equal variances) was used and statistical significance was depicted by asterisks (*): * p <0.05, ** p <0.01, *** p <0.001, **** p <0.0001.

.
